# Exploring how Japanese pharmacists provide reasonable accommodations for persons with intellectual disabilities

**DOI:** 10.1186/s12913-025-13035-7

**Published:** 2025-07-03

**Authors:** Masaki Shoji, Rintaro Imafuku, Mei Mizomoto, Mitsuko Onda

**Affiliations:** 1 Department of Social and Administrative Pharmacy, Osaka Medical and Pharmaceutical University, 4-20-1, Nasahara, Takatsuki City, Osaka 569-1094 Japan; 2 Nursing Research Promotion Center, Graduate School of Nursing, Nagoya City University, 1 Kawasumi, Mizuho-cho, Mizuho-ku, Nagoya City, Aichi 467-8601 Japan; 3 SHINTANI Clinic of Psychosomatic Medicine, Nishiura 87, Hironocho , Uji City, Kyoto 611-0031 Japan

**Keywords:** Intellectual disability, Pharmacist, Communication, Reasonable accommodation

## Abstract

**Background:**

There has been limited progress in research on reasonable accommodations for persons with mild/moderate intellectual disabilities (ID) who are able to live independently but have struggles. This study aims to qualitatively investigate how pharmacists provide reasonable accommodations when communicating with people with ID.

**Methods:**

Between October 2023 and March 2024, semi-structured interviews were conducted with 11 hospital or community pharmacists. They were mainly asked about how they have dealt with people with ID. Interviews were conducted either in person or online, each lasting 30–60 min. Data were analyzed using a thematic analysis approach.

**Results:**

This study identified three main themes regarding the pharmacists’ reasonable accommodation practices: “comprehension aids,” “reminders,” and “structural accommodations.” Specifically, to help the individuals better understand, pharmacists tried to use simple expressions and visual information, including pictures and symbols, and to adjust their speaking speed and volume. As reminder strategies, they provided supplementary handwritten information, highlighted the main points, and use of post-its. Furthermore, they illustrated the medication sequence as a structural accommodation strategy.

**Conclusions:**

The study suggests that pharmacists have employed several strategies to offer reasonable accommodations, aiming to build and maintain better relationships with people with ID and to promote these individuals’ understanding of medications, adherence, and treatment safety.

## Backgrounds

Intellectual disability is one of the most common neurodevelopmental disorders in the DSM-5 (Diagnostic and Statistical Manual of Mental Disorders, Fifth Edition). It is estimated that about 1 million people in Japan are affected by intellectual disability since the revision of the DSM in 2013, intellectual disability in adulthood have been attracting greater attention. Intellectual disability result in deficits in both intellectual and adaptive functions, limiting the ability to think logically and abstractly, plan, communicate in the community, and live independently. The severity of the disability varies greatly from person to person, and although people with mild to moderate intellectual disability can often lead their daily lives without the assistance of a caregiver, they often face difficulties due to their limited functions. It is estimated that people with intellectual disability have higher medical demands and are prescribed medications more regularly, about 4 times more than those with typical development [1]. Furthermore, it has been reported that elderly people with intellectual disability are more prone to polypharmacy [2–4] and that an intellectual disability is a background factor for inappropriate prescribing [3]. Medical communication encompasses much information that people with intellectual disability find difficult to understand, such as explanations of medical conditions that are highly abstract and explanations about medications that require unique manipulation and planning. Smith et al. point out that people with intellectual disability lack an understanding of their own drug therapies [5]. In particular, mistakes or misunderstandings regarding medication lead directly to health problems, so care must be taken in the way information is conveyed. A limited number of studies have reported on pharmacists’ interventions with people with intellectual disability [6, 7] and highlighted the challenges in evaluating the impact of these interventions [7]. Lee et al. indicated that there are seven major types of interventions by pharmacists for people with intellectual disability in the world: medication usage, cost/effectiveness, drug related problems, drug related interventions, caregiver and healthcare team satisfaction, secondary symptoms, and education/knowledge [7]. In Japan, most research to date on accommodations for people with intellectual disability has focused on how nursing home staff administer medications to people with severe disability [4]. On the other hand, there has been limited progress in research on reasonable accommodations for people with mild/moderate intellectual disability who are able to live independently but have struggles. In 2015, the Osaka Hand in Hand Inclusion Society [8] created “Guidelines for Providing Easy-to-understand Information” under the Ministry of Health, Labor and Welfare’s FY2015 Comprehensive Welfare Promotion Project for Persons with Disabilities (Table 1) [9], but the actual utilization in the medical field has not been examined. Therefore, the purpose of this study was to explore issues of health communication between pharmacists and people with intellectual disability. Specifically, the main research question is: How are pharmacists providing reasonable accommodations when conveying pharmaceutical information to people with intellectual disability?

**Table 1:** Guidelines for providing easy-to-understand information (selected excerpts)

Writing Expressions
Use plain expressions
Include specific information
Delete non-essential information as much as possible.
No metaphors, allusions, or personification.
Don’t use double negatives.
Do not use demonstrative words, such as “this” or “that,” that refer back to other words that appear earlier in the text, because this can confuse persons with disabilities. Instead, repeat the original word and) phrase for emphasis and clarity.
Describe what will happen in the chronological order in which it will happen, so the reader will not have to put any effort into interpreting or reconstructing the timeline.
The time should be written in 12-hour format, not in 24-hour format.
Visual Expressions
The font size should be at least 12 point.
Separate text into meaningful units.
Break lines with an awareness of the cohesiveness of information.
Layout tips
Use photos and illustrations that show the content of the story.
Emphasize the important information or keywords by coloring, bolding, enclosing, etc.
Things to keep in mind when providing explanations
Consider the characteristics of the person’s reading and listening comprehension skills
Respect the age of the subject and use age-appropriate language.

## Definitions

### Intellectual disability

This study adopts the DSM-5’s definition of intellectual disability as neurodevelopmental disorders that begin in childhood and are characterized by intellectual difficulties as well as difficulties in conceptual, social, and practical areas of living. The DSM-5 diagnosis of intellectual disability requires the satisfaction of three criteria:

1. Deficits in intellectual functioning—“reasoning, problem solving, planning, abstract thinking, judgment, academic learning, and learning from experience”—confirmed by clinical evaluation and individualized standard IQ testing.
2. Deficits in adaptive functioning that significantly hamper conforming to developmental and sociocultural standards for the individual’s independence and ability to meet their social responsibility; and.
3. The onset of these deficits during childhood.

However, it might be difficult in the pharmacy setting to determine whether the person has an intellectual disability.

In this study, we asked the pharmacists not to think too seriously about whether the person truly had an intellectual disability, but to talk about how they had responded to a person whom they subjectively judged, based on their own observations while providing medication instructions, to have had difficulty understanding the instructions.

### Reasonable accommodation

Referring to the Convention on the Rights of Persons with Disabilities, this study defined “Reasonable accommodations” as “necessary and appropriate modifications and adjustments to ensure that people with disabilities enjoy or exercise all human rights and fundamental freedoms on the basis of equality with others, which are necessary in particular cases and which are not unbalancing or unduly burdensome.”

## Methods

Semi-structured interviews were conducted with 11 pharmacists working in pharmacies or hospitals in Osaka, Japan, between October 2023 and March 2024. Interviews were conducted either in person or online via Zoom, and each interview lasted 30–60 min. All interviewees were interviewed once.

Purposive sampling was used in this study. The selection criterions for the interviewees were “pharmacy or hospital pharmacists with experience in dealing with people with intellectual and developmental disabilities”; pharmacists who had been out of clinical practice for more than 3 years were excluded. In the interview, the participants were asked about their experiences of dealing with people with intellectual disability. The first author of this article conducted the interviews. The author is a pharmacist, holds a PhD, and has experience in qualitative research. Of the 11 interviewees, six were acquainted with the first author at the request stage and five were not. The interviewees received a written explanation of the purpose and outline of this study from the first author, either in person or by e-mail. The interviews were recorded with the interviewees’ consent.

This study adopted an interpretivist paradigm to explore pharmacists’ experiences in supporting individuals with intellectual disabilities. Qualitative data in this study were analyzed inductively using Braun and Clarke’s thematic analysis, which offers a six-step guide for analysis [10]. Through a thematic analysis of interview transcripts, we aimed to understand how pharmacists construct meaning in their interactions. NVivo R1.6 (QSR International, Australia) was used for data analysis. The details of interview questions are shown in Table 2.

**Table 2:** Interview guide

Questions	Contents
Introduction	Career, years of service
Degree of involvement with developmental disorder children prior to employment	How have you interacted with children with intellectual or developmental disorder? What did you feel at the time?
Opportunities to interact with patients with intellectual or developmental disorder as a pharmacist	How often do you have the opportunity to see patients with suspected developmental or intellectual disabilities?
Circumstances leading to the provision of reasonable accommodations	How the pharmacist observes and evaluates the patient and comes to the conclusion that reasonable accommodations need to be provided
Reasonable accommodations provided during medication instruction	What kinds of accommodations are provided and how the patient reacts Have you experienced problems with patients? Are there any easy-to-understand materials for people with intellectual and developmental disabilities in your pharmacy.
Issues in pharmacy education	Experience with university or privately provided education on how to treat patients with intellectual or developmental disorder. What advice would you give to future generations on how to deal with patients with intellectual or developmental disorder?

### Ethical considerations

This study was conducted in compliance with the Declaration of Helsinki and the “Ethical Guidelines for Medical Research in Humans.” Approval was obtained from the Osaka Medical and Pharmaceutical University Faculty of Medicine Research Ethics Review Committee (Approval No. 2023-062). All participants signed an informed consent statement prior to participation in the study.

## Results

All 11 were interviewed, and none withdrew their consent during or after the interview. The average length of employment for the research participants was 8.5 years. Three worked in hospitals and eight in pharmacies. Five of the eleven had prior employment experience playing with or helping classmates and others with developmental or intellectual disabilities on a one-on-one basis (Table 3).

**Table 3:** Interviewee characteristics

Interviewee	Years of service	Employment	Interview	Degree of involvement with intellectual or developmental disorder prior to employment^a^
A	3	H.P.	Face to face	Close
B	8	C.P.	Face to face	Moderate
C	3	H.P.	Online	Not much
D	3	C.P.	Online	Close
E	34	H.P.	Face to face	Moderate
F	7	C.P.	Group (F&G)	Not much
G	3	C.P.	Group (F&G)	Moderate
H	7	C.P.	Group (H&I)	Close
I	8	C.P.	Group (H&I)	Close
J	6	C.P.	Face to face	Close
K	12	C.P.	Face to face	Moderate

*H.P*. Hospital Pharmacist, *C.P.* Community Pharmacist

^a^Close: Have played with or helped a child with a disability on a one-on-one or similar basis for a period of time. Moderate: Have talked to or played with a child with a disability as a member of the class on several occasions. Not Much: Knew there was a child with a disability at school, but did not have any contact with him or her at that time or since

The number of initial codes was 298, and the content was broadly divided into decisions leading up to the provision of considerations and considerations implemented at the time of medication instruction.

### The process leading to accommodations

**Fig. 1:**
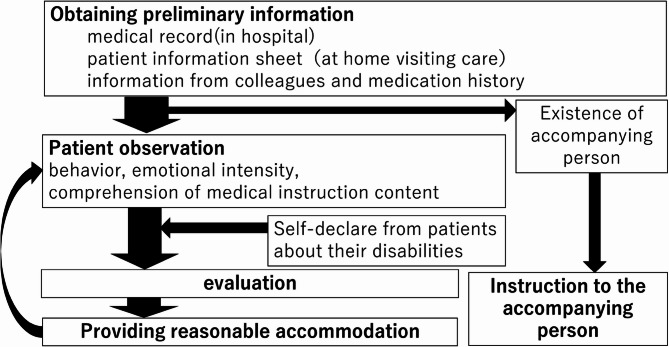
Process leading to the provision of reasonable accommodations

Figure 1 illustrates the process leading up to providing the accommodations. Before providing guidance, the pharmacist checks the patient’s information, such as the patient’s medical records, medication history, and patient information sheet at the time of in-home medical care management guidance. The pharmacist then observes the person, and if the person is accompanied by a companion, the pharmacist often provides guidance primarily to the companion. If the person is unaccompanied, the patient’s behavior after entering the pharmacy, emotional ups and downs, and level of understanding during medication instruction are observed, and the need for accommodations evaluated, and the necessary accommodations are then implemented. If the person self-reports his or her disability, then it is easier to provide the appropriate accommodation.

### Accommodations provided by pharmacists

**Fig. 2:**
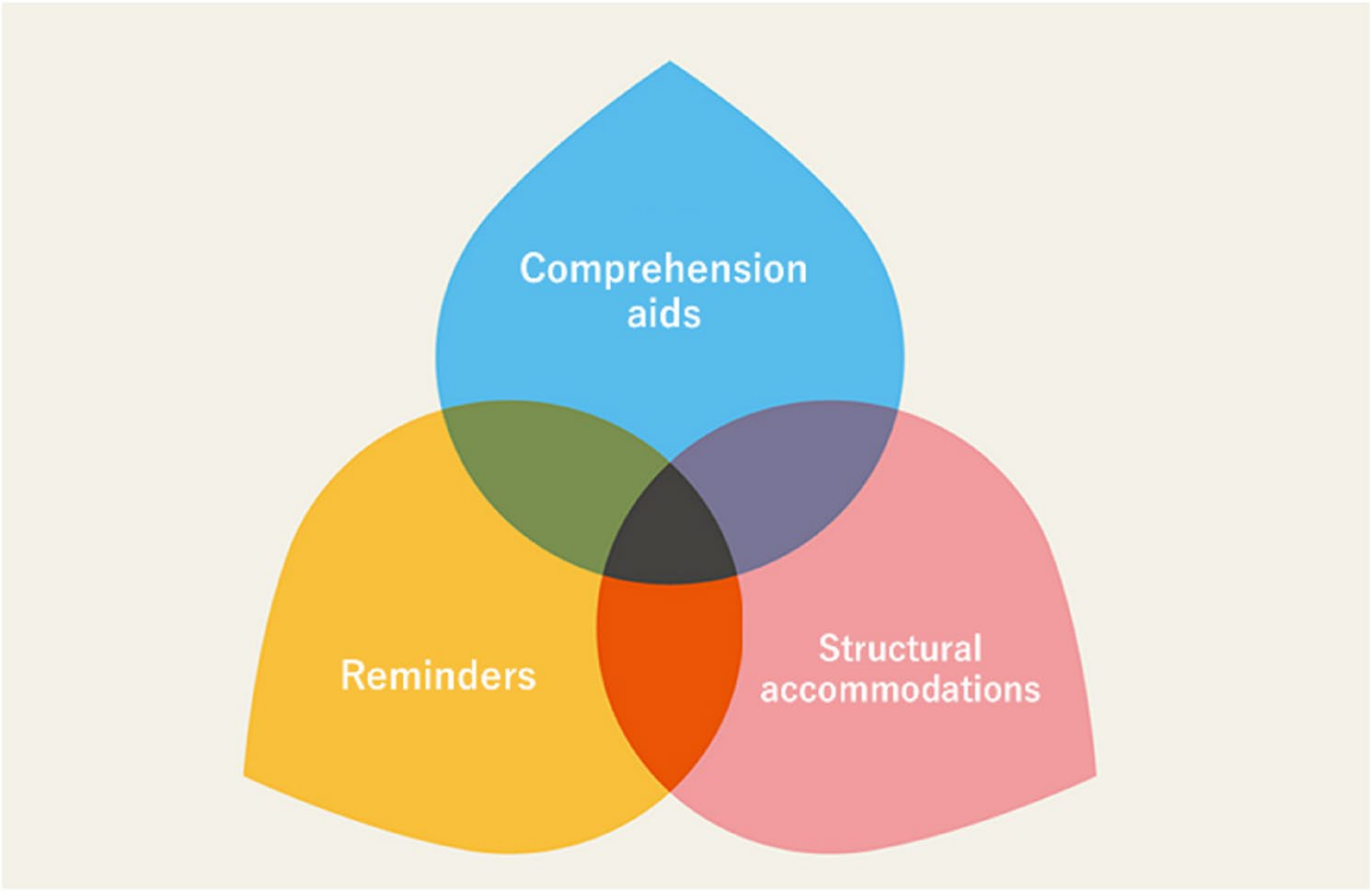
Reasonable accommodations for persons with intellectual disability provided by pharmacists during medical consultations

Based on the responses obtained from the interviews, the pharmacists’ reasonable accommodations for people with intellectual and developmental disabilities were broadly classified into “comprehension aids,” “reminders,” and “structural accommodation” (Fig. 2).

#### Theme 1: Comprehension aids

The most frequently mentioned consideration that pharmacists make is a change in the way they communicate. Several pharmacists mentioned the importance of adjusting speaking speed and volume, and using simple language. Some also emphasized the need for “Specific instructions” and avoiding ambiguous expressions. Others highlighted the necessity of “Repetition” of same explanation to ensure individuals understand (Table 4).

**Table 4:** Comprehension aids

Sub-theme	Quotations
Use simple language	*I speak in simple language*,* basically. I do the same with all my patients*,* though.* (PF)
Take enough time	*Basically the same with all patients*,* but with simpler expressions and more time spent. I persist until they are satisfied.*(PF)
Specific instructions	*If a patient repeatedly asks me when he or she should take their medication because of anxiety*,* I think it is better to be clear about the specifics*,* such as how many times and how many hours apart*,* or if it is a sleeping pill*,* what time to take it by*,* or other rules.* (PC)
Adjust speaking speed and volume	*I would first slow down the speed of my speech. If the patient is young*,* I normally talk quite fast*,* but if I feel that the patient is not following me at all*,* I think*,* “Oh*,* maybe this patient needs a little more time to understand*,*” and I slow down or repeat my words slowly.*(PB)
Use of pictures and symbols	*We drew an illustration on a medicine bag. For “after breakfast*,*” I drew a picture that looked like a sunrise. For “after lunch*,*” a picture of the sun at high noon*,* and for “after dinner*,*” a picture of the moon*. (PE)
Adjust the amount of information	*I tried not to give too much information*,* and I was very conscious of that*. (PD)
Repetition	*Even after I explain something and the patient understands it*,* he/she often asks the same question again. I have the impression that many times they ask the same questions over and over again*,* and I have to spend a little time explaining things to them.* (PC)

#### Theme 2: Reminders

This theme included ways to remind people of the need to prevent mistakes when taking medications after the person returns home. Several pharmacists talked about providing supplemental handwritten advice, highlighting important points during explanations, and using post-its to prevent the person from forgetting things (Table 5).

**Table 5:** Reminders

Sub-theme	Quotations
Add supplemental handwritten advice	*I would write notes*,* or rewrite things on the medicine information or medicine bags in a way that was easy to understand*,* or whatever I could do. It was something that didn’t take much time and effort*,* and I would do things like that for them.* (PJ)
Highlighting	*Many of the pharmacists in my workplace draw a circle or something to point out the cautions.* (PB)
Use of post-its	*I might tell them to put a post-it on it. If there was a patient who always forgot to bring his or her medication book*,* then I would say*,* like*,* “You’re bringing your consultation ticket*,* right? Why don’t you put a post-it there?”* (PB)

#### Theme 3: Structural accommodations

Under the theme “Structural accommodations,” the pharmacists mentioned methods of avoiding problems such as persons making mistakes when taking their medications by using “Single-dose packaging”. Also, the subtheme “Sequence visualization” was mentioned to prevent problems related to waiting time (Table 6).

**Table 6:** Structural accommodations

Sub-theme	Quotations
Single-dose packaging	*If the patient seemed a little anxious*,* the medicines are in single-dose packages*,* so I would say*,* like*,* “It’s in a single package which says ‘after breakfast*,*’ so you don’t have to remember.” to them.* (PA)
Sequence visualization	*The receptionists are always discussing how to make the patient feel at ease. For example*,* if there is a patient who is not good at waiting their turn*,* they announce*,* “XX patients are waiting*,*” “You are next*,*” like that.* (PE)
Asking physician for single-dose packaging instructions	*If I find a patient who is not likely to able to understand how to take his/her medication*,* then I recommend single packaging. Even some ENT medications in single-dose packaging. Then*,* I call the doctor and say something like*,* “I think this patient should continue to receive single-dose packaged medication.” Then the doctor will give the order for that next time.* (PB)

## Discussion

This study is the first attempt in Japan to explore the reasonable accommodations that pharmacists provided for people they think have intellectual disabilities. Although the pharmacists had difficulty determining whether the patient in front of them had an intellectual disability, it was evident that they sensed the need for consideration during the conversation and practiced various communication techniques.

Heimerl et al. noted that pharmacists have difficulty in determining the presence or absence of cognitive impairment in the dementia patients they deal with [11], which is common to intellectual capacity impairment. Dooley et al. also point out the difficulty of balancing communication considerations for the patient with dementia himself/herself and the amount of communication with his/her companion [12], which is also a common challenge with intellectual disability. This study identified three main themes regarding the reasonable accommodations provided by the pharmacists: “comprehension aids,” “reminders,” and “structural accommodations.”

The theme “Comprehension aids” is similar to the previous report on “plain writing.” Information being accessible and textual information being written in an “understandable” form can be empowering for people with intellectual disabilities [13–15]. For example, Mencap, a British organization dedicated to helping people with intellectual disabilities, is a leading explorer and promoter of easy-to-understand information dissemination methods. Mencap publishes a variety of content on its website to facilitate communication [16]. As mentioned above, in Japan, we have “Guidelines for Providing Easy-to-understand Information”. Although the guidelines mainly focus on the communication of information in writing, some of the items listed, such as “do not use difficult words,” “include specific information,” “delete information that is not necessary as much as possible,” and “use pictures and illustrations,” were recognized as equivalent to the contents in the statements of the interviewees in our survey. However, although photographs and illustrations are widely used, some reports are skeptical as to whether they really facilitate understanding for people with intellectual disability [14, 15], because the meaning of a picture or symbol is not always understood as intended by the writer, suggesting that further studies are needed. It is also noteworthy that the interviews in our study were mainly related to accommodations in providing oral medication guidance, and thus included accommodations that are not included in the guidelines, such as “take enough time,” “adjust speaking speed and volume,” and “repetition.” On the other hand, as many pharmacists have mentioned, specialized instructional media designed for people with intellectual disability are generally not used in pharmacies and hospitals. We believe that the development of specialized instructional media, such as explanations written in more plain language and a larger font size, will be needed going forward.

Regarding the “reminders” strategy, “Highlighting” corresponds to one of the items listed in the guidelines: “The most important information or keywords should be coordinated by color coding, bolding, or enclosure.” These accommodations could help prevent misunderstandings about the amount of medication taken per dose or the number of doses per day. In addition, if the drug information form contains lengthy or difficult-to-understand explanations, people may skip over the text. Therefore, highlighting important information can help individuals understand by removing as much of the less necessary information as possible.

Regarding the “Structural considerations”, the “Single-dose packaging” service, in which medicines are packaged in plastic bags and provided to patients at each dose, is effective for keeping people from making mistakes or forgetting to take their medicines. On the surface of the plastic bag, the dosage, date and time of administration, name, and other information can be printed. Single-dose packages have long been suggested to improve adherence, especially for elderly people [17]. Sato et al. pointed out the high rate of dispensing of single-dose packaging in neuropsychiatric departments and considered the reasons for this, such as the long duration of drug therapy and the difficulty of handling tablets and capsules, depending on the disease [18]. However, our survey suggests that the high frequency of single-dose packaging in mental and neurological disorders may also be aimed at preventing decreased adherence due to intellectual and developmental disabilities.

We recognize the following limitations of this study. First, it is difficult to identify mild intellectual disability in pharmacies (or even in specialized institutions), and the presence of such disability is determined subjectively through observation and evaluation by the pharmacist. Second, intellectual and developmental disabilities manifest themselves in very different ways depending on the presence of complications or the individuals’ environment, and it is only after multiple interviews that some of the unevenness in the individuals’ abilities can be ascertained. Therefore, it is difficult to say that the findings of this study are universal. Despite the above limitations, we believe that this study will be a valuable resource, as there have been few studies examining how to provide accommodations to people with intellectual and developmental disabilities from the standpoint of providing information on pharmacotherapy.

## Conclusions

The study suggests that pharmacists have employed three categories of strategies to offer reasonable accommodations; Comprehension aids, Reminders, Structural accommodations. These strategies aiming to build and maintain better relationships with people with intellectual disabilities and to promote these individuals’ understanding of medications, adherence, and treatment safety. For further research, it would be worthwhile to investigate individuals’ perspectives on the reasonable accommodations provided by the pharmacists.

## Data Availability

The datasets generated and analyzed during the current study are available from the corresponding author on reasonable request.
